# Some Peculiar Cases of Dental Resorption

**Published:** 1902-07

**Authors:** Otto E. Inglis

**Affiliations:** Philadelphia


### SOME PECULIAR CASES OF DENTAL RESORPTION.[1]

BY OTTO E. INGLIS, D.D.S., PHILADELPHIA.[2]

It is a generally accepted fact that resorption of tissue is effected through the agency of cells belonging to the connective-tissue group and commonly called "giant-cells." These are large cells, either mononuclear or multinucleated, found to be capable of excreting a substance which liquefies or detaches small particles of even solid tissues, such as bone or even enamel. The resulting chemical product or particle is either washed away by the lymph-stream or taken into the body of the giant-cell and borne away.

These multinucleated cells are found in granulation-tissue, which in turn is in some form almost invariably found in apposition with surfaces or tissues undergoing resorption. It has been suggested that the solvent excreted is lactic acid, though there seems to be no demonstration of this fact.

Theoretically, acid sodium phosphate would seem to be more likely to be the solvent, as surfaces undergoing resorption are eroded, not decalcified. The writer has endeavored in vain to get an acid reaction with litmus paper touched to the granulations, though numerous efforts have been made. It is quite possible, however, that any acid present may be masked by the general alkaline reaction of the blood plasma, while the giant-cell itself, excreting such an acid against a tissue, may accomplish its physiological object.

Resorption of deciduous roots occurs as a physiological process, while resorption of permanent roots is classed as pathological. There are also records of a few cases of resorption of the crown dentine of permanent teeth, presumably through the agency of giant-cells in the dental pulp.

In all cases it seems possible to refer the resorption to irritation with the production of a mild grade of non-purulent inflammation as a cause. There seems evidence enough to warrant this assumption.

Pus formation retards or prevents resorption presumably

---

[1] Read before the Academy of Stomatology, November 26, 1901.

[2] Special Lecturer on Dental Pathology and Therapeutics in the Philadelphia Dental College.

through neutralization of the acid product of the giant-cells, the reaction of pus being alkaline. When pus formation is in abeyance, granulations spring up.

In Gaskill's case of dentine resorption[1] by the pulp the vascular alteration in the pulp was shown by the pink color transmitted through the enamel, this being evidence of a condition of advanced hyperæmia or of mild inflammation.

Resorption of cementum and dentine occurs in resorption of deciduous roots, and is produced by the resorbent organ exterior to the tooth. In a certain number of specimens of loose deciduous crowns there may be found after extraction bays of resorption in the dentine of the crown. In a case of extreme excavation the entire dentine of the crown was removed by the resorbent organ, the enamel alone remaining.

In a third case the dentine was largely removed and the tooth perforated upon the mesial side from within by the resorbent organ. A diagnosis of suffusion from hyperæmia had been made, but upon rapid extraction the pulp was found as a large soft mass, but in a vital state within the crown; that is, the erstwhile pulp had become a resorbent organ. All appearance of suffusion disappeared with its removal from the crown concavity, and the tiny mesial opening was discovered. There were no signs of caries whatsoever. This case practically corresponds to that of Gaskill's, but adds the important fact that enamel might readily be resorbed by granulation-tissue from within. That in Gaskill's case is described as crushed in.

In a left lower deciduous lateral incisor, standing labially to the permanent left lower lateral incisor, extraction revealed a hyperæmic condition of the pulp for a length of one-quarter of an inch from the apical end of the root, which was but slightly resorbed. The root end was transparent and the hyperæmia visible as a clearly defined pink streak.

While the pulp may become involved in the resorbent organ and become a part of it, it does not of necessity follow that it does so under all circumstances, as some specimens show a condition the reverse of that previously mentioned. Tomes maintains that resorption of deciduous roots is a vital act not dependent upon the pressure of the advancing permanent tooth.

---

[1] Proceedings Academy of Stomatology, 1895.

If one observes, however, the numerous examples of resorption occurring at the pressure point, it will seem hard to accept the theory even when resorption does occur without a tooth near by to cause pressure. Deciduous cuspids are notoriously retained late when the permanent cuspid erupts late, or anteriorly or posteriorly to the deciduous tooth. It is true that these deciduous cuspids are often later lost by resorption, but in all probability they have been somewhat affected by resorptive action during the descent of the cuspid. In a mouth recently seen eight deciduous molars were in place at about twenty-one years of age. There was no mechanical impediment to their shedding.

In the resorption of the roots of the permanent teeth aseptic irritation seems to be the proximate cause for all devitalized teeth, and where the pulp is vital it is not always possible to say that irritation is not still the cause. For example, some such teeth are in malocclusion, some in pyorrhœal conditions, and in others, again, marginal gum resorption or pulp irritation is going on.

The existence of a dyscrasia is also recognized. A patient of mine lost a right lower molar by resorption after arsenic was applied to the pulp. I naturally connected the resulting pericementitis with a possible passage of arsenic through the foramen, and made a record of the case. Since then I have extracted two resorbed supernumerary molars, and have discovered that the lingual root of an upper molar is partly resorbed. The tooth is still in the mouth, but the lingual root has lost the alveolar process upon its lingual aspect by phagedenic pericementitis and resorption, a fact which enabled me to discover the loss of its apical third. The patient is a neurasthenic.

Among the specific recognized causes of resorption in permanent teeth are the following: Chronic abscess (temporarily in abeyance), plantations, protruding root-fillings or broaches, looseness, and pressure. A certain number of cases are due to blows and malocclusion, a few to calculus and pyorrhœa alveolaris.

I have a specimen which shows a curious combination of resorption and calculus formation from a case of prolonged chronic abscess. The resorption doubtless was due to the formation of granulation-tissue during such time as pus formation was absent or not active; or, possibly, the inflammation of the pericementum at a point contiguous to the area of pus formation might account for the resorption.

The frequent occurrence of resorption in connection with chronic abscess of long duration inclines me to believe that the former is the true explanation, especially as I have seen granulations *in situ* after extraction. It is, of course, possible that the pyogenic germs may eventually be shown to have some erosive action. At present such a view cannot be accepted without a demonstration; indeed, Green[1] states that an ivory peg may lie in pus for weeks without visible effect, while it undergoes resorption if aseptically buried in the tissues.

A transient patient from the practice of Professor Eames, of Boston, presented an unusual resorption upon the lingual surfaces of four lower incisors, calculus being the exciting cause. The pulps were not exposed.

Resorptions after plantations are explained upon like grounds of formation of granulation-tissue, bony ankylosis being later brought about by a better tolerance and a constructive activity of bone-cells. The peculiar generally distributed resorption in cases of exfoliation after plantation is evidence of a general pericementitis.

Curious resorptions of roots from about gutta-percha root-fillings, cotton root-fillings, and protruding broaches are quite common.

A root may be perforated from the side to the canal, or even from side to side, by resorptive action. I have a specimen which shows a remarkable case of resorption of permanent roots simulating resorption of deciduous roots and due to the same cause,—viz., pressure of an advancing tooth. The history was that of indefinable neuralgic pain about the third upper molar, necessitating extraction. When removed, the buccal roots being absent, it was thought that a fracture had occurred. An attempt at their removal resulted in the dislodgement of a fourth or supernumerary molar the crown of which exactly fitted the area of resorption at the cervical third of the buccal roots.

Other cases of resorption of permanent roots by advancing crowns of impacted teeth have been discovered by means of the Röntgen ray, and after extraction for relief of pain.

In some cases of impacted teeth even the enamel may suffer marked resorption, as in the case of a cuspid taken from the jaw

---

[1] Pathology and Morbid Anatomy.

of a lady sixty-five years of age. The tooth slowly erupting was upon one side of the gum and a gold plate upon the other. The resultant tumefaction was natural. Ulceration occurred; the crown came into relation with the oral fluids; granulations formed upon the under surface of the tumefaction, with the result that resorption, but no caries, supervened, or, indeed, may have occurred previously, as it was present and obvious to instrumental diagnosis for months before extraction.

Miller, recently, and Cryer, previously, have noted the condition of enamel resorption.

Another specimen in the writer's possession shows a circumscribed bay-like resorption of enamel and dentine.

The term resorption having been objected to by a prominent friend as not applicable to enamel, it may perhaps be said, in defence of the term, that it seems better than the term "absorption," for though not deposited by mesodermic tissue, enamel is removed in the same manner as tissues of mesoblastic origin, and to be exigent in this matter would be to necessitate the abandonment of use of the word in connection with nerve-tissue as well.

